# Clustering Analysis of Seismicity in the Anatolian Region with Implications for Seismic Hazard

**DOI:** 10.3390/e25060835

**Published:** 2023-05-23

**Authors:** Davide Zaccagnino, Luciano Telesca, Onur Tan, Carlo Doglioni

**Affiliations:** 1 Department of Earth Sciences, Sapienza University of Rome, 00185 Roma, Italy; carlo.doglioni@uniroma1.it; 2 Institute of Methodologies for Environmental Analysis, National Research Council, 85050 Tito, Italy; luciano.telesca@imaa.cnr.it; 3 Department of Geophysical Engineering, Faculty of Engineering, Istanbul University-Cerrahpaşa, Istanbul 34320, Turkey; onur.tan@iuc.edu.tr; 4 National Institute of Geophysics and Volcanology, 00143 Roma, Italy

**Keywords:** clustering coefficients, b-value, maximum magnitude, seismogenic potential

## Abstract

The Anatolian region is one of the most seismically active tectonic settings in the world. Here, we perform a clustering analysis of Turkish seismicity using an updated version of the Turkish Homogenized Earthquake Catalogue (TURHEC), which contains the recent developments of the still ongoing Kahramanmaraş seismic sequence. We show that some statistical properties of seismic activity are related to the regional seismogenic potential. Mapping the local and global coefficients of variation of inter-event times of crustal seismicity which occurred during the last three decades, we find that territories prone to major seismic events during the last century usually host globally clustered and locally Poissonian seismic activity. We suggest that regions with seismicity associated with higher values of the global coefficient of variation of inter-event times, C${}_{V}$, are likely to be more prone to hosting large earthquakes in the near future than other regions characterized by lower values, if their largest seismic events have the same magnitude. If our hypothesis is confirmed, clustering properties should be considered as a possible additional information source for the assessment of seismic hazard. We also find positive correlations between global clustering properties, the maximum magnitude and the seismic rate, while the b-value of the Gutenberg–Richter law is weakly correlated with them. Finally, we identify possible changes in such parameters before and during the 2023 Kahramanmaraş seismic sequence.

## 1. Introduction

### 1.1. Current State of Knowledge

Earthquakes are the final outcome of long-lasting processes of energy accumulation in the brittle crust due to the action of tectonic forces [1]. The nucleation of seismic events starts as soon as the local differential stress overcomes friction and fracture resistance; however, the dynamics of rupture propagation and arrest, as well as the spatial and temporal evolution of seismicity, depend on both the physical properties and the detailed structural organization of the fault systems, e.g., [2]. Therefore, both on- and off-fault rheology and boundary conditions play a role in shaping seismic sequences [3]. Rheology is likely to control the mechanism of stress accumulation and drop, i.e., how tectonic strain is spatially accommodated and released via a wide range of possible seismic dynamics, e.g., [4,5,6]. For instance, slow slip events tend to be nucleated within weak interfaces along the shallow section of subduction zones, large megathrust events occur in the locked segments close to the trench and aseismic creep takes place where stress is continuously dissipated by spread ductile deformations. On the other hand, long-range interactions are mainly responsible for the temporal evolution of seismicity [7,8,9]; for this reason, statistical patterns of earthquake activity preceding major seismic events have been widely investigated, e.g., [10,11,12,13]. Stress transfer due to preceding events and strain arrangement within crustal volumes provide the ultimate conditions for the dynamic propagation of fracture during the coseismic phase and for the destabilization of fault patches during seismic sequences. Such a complex pattern of interactions leads to both long- and short-term clustering of seismicity over several spatial scales, e.g., [14]. For this reason, clustering features of seismic activity have been extensively studied using different approaches, ranging from classical statistical analysis to artificial intelligence, both in the laboratory and in real fault systems [15,16,17,18].

### 1.2. Aim of the Work

Collective parameters can be extremely useful for characterizing the clustering properties of seismicity [19,20,21,22]; moreover, more recently, it has been suggested that they can be related to the behavior of seismogenetic sources at a regional scale [23]. Therefore, they may be of interest to infer the seismogenic potential of still poorly investigated areas. In this work, we analyze Anatolian seismicity since 1990, considering the major (M${}_{w}\geq$ 5.5) events since 1905 listed in the Turkish Homogenized Earthquake Catalogue (TURHEC) [24,25]. Particular attention is devoted to the southeastern region of Turkey, recently affected by the Kahramanmaraş seismic sequence. We mostly focus on its statistical properties, in particular, long-term global clustering, and the scaling exponent of the frequency-size Gutenberg–Richter law.

## 2. Methods

In this work, we only consider seismic events contained in the TURHEC seismic catalogue which occurred from 1 January 1990 to 27 February 2023 from latitude 34${}^{\circ }$ to 44${}^{\circ }$ N and between longitude 25${}^{\circ }$ and 46${}^{\circ }$ E. In addition, seismic events are only considered if their depth is shallower than 30 km and their size is above the completeness magnitude (compare with the next paragraph). We also consider the M${}_{w}$ 5.5+ earthquakes that occurred from 1905 to 2023 in the same region reported in the same catalogue. In our analysis, we divide the Anatolian region into rectangular contiguous areas. The number of parts is chosen to allow a reliable assessment of the statistical properties of seismicity according to the different sources of uncertainty and their variation along the catalogue. For the assessment of the b-value, a 15 × 6 grid, along longitude and latitude, respectively, is used to guarantee reliable statistical resultswhile a 30 × 15 grid is applied otherwise.

### 2.1. Catalogue Completeness

In this investigation, only earthquakes above the completeness magnitude are considered. We apply the Wiemer–Wyss method [26] and add a correction of +0.2 magnitude units, as suggested in [27]. The completeness magnitude is computed for samples of one thousand earthquakes each in order to take into account the different stages of seismic activity usually associated with variable catalogue completeness.

### 2.2. Coefficients of Variation

The global coefficient of variation of inter-event times, $C_{V}$, defined by [28]

(1) $$C_{V}=\frac{\sigma_{\Delta T}}{\langle \Delta T\rangle }$$

where 〈ΔT〉 represents the mean value of the inter-event time and $\sigma_{\Delta T}$ is its standard deviation, is applied to study the temporal clustering of seismicity. If $C_{V}<1$, the dynamics is regular; in contrast, if $C_{V}>1$, the dynamics is clustered. The condition $C_{V}=1$ stands for a completely random Poisson process [29]. Conversely, the local coefficient of variation, $L_{V}$ defined by [30]

(2) $$L_{V}=\frac{3}{N-1}\sum_{i=1}^{N-1}\frac{{\left(T_{i}-T_{i+1}\right)}^{2}}{{\left(T_{i}+T_{i+1}\right)}^{2}}$$

is routinely utilized for quantifying the local variability of the inter-event time series. The meaning of the values of $L_{V}$ is the same as $C_{V}$.

### 2.3. b-Value

The Tinti–Mulargia [31] and the maximum likelihood Aki–Utsu [32] methods are applied for the estimation of the b-value of the Gutenberg–Richter law. The first technique performs well in the case of limited catalogues; moreover, it takes into account the magnitude of binning, while the second technique is a standard method with widespread applicability in the case of quite large catalogues and magnitudes ranging at least over three bins. In order to utilize it, $\langle M_{w}\rangle$ and the threshold (minimum completeness) magnitude $M_{wc}$ are required.

The first is obtained by the definition of the arithmetic mean of the *N* magnitudes in the catalogue, while the second is estimated using the Wiemer–Wyss method [26], with an additional correction of +0.2 magnitude units, as described above.

## 3. Analysis and Results

**Figure 1:**
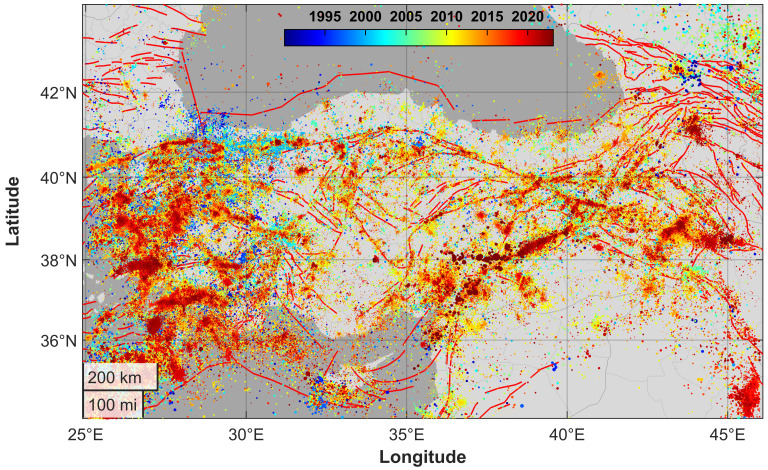
Map of seismicity in the Anatolian region. Each point represents an earthquake (TURHEC Catalogue, 1990–2023). Seismic events with epicenters located between 25 and 46${}^{\circ }$ E of longitude and 34 and 44${}^{\circ }$ N of latitude and a hypocenter shallower than 30 km. Red lines represent mapped active faults (data from GEM Global Active Faults database).

Since 1990, seismicity in Turkey has mainly taken place offshore in the Aegean Sea and along different segments of the Northern and, more recently, of the Eastern Anatolian fault systems (Figure 1).

**Figure 2:**
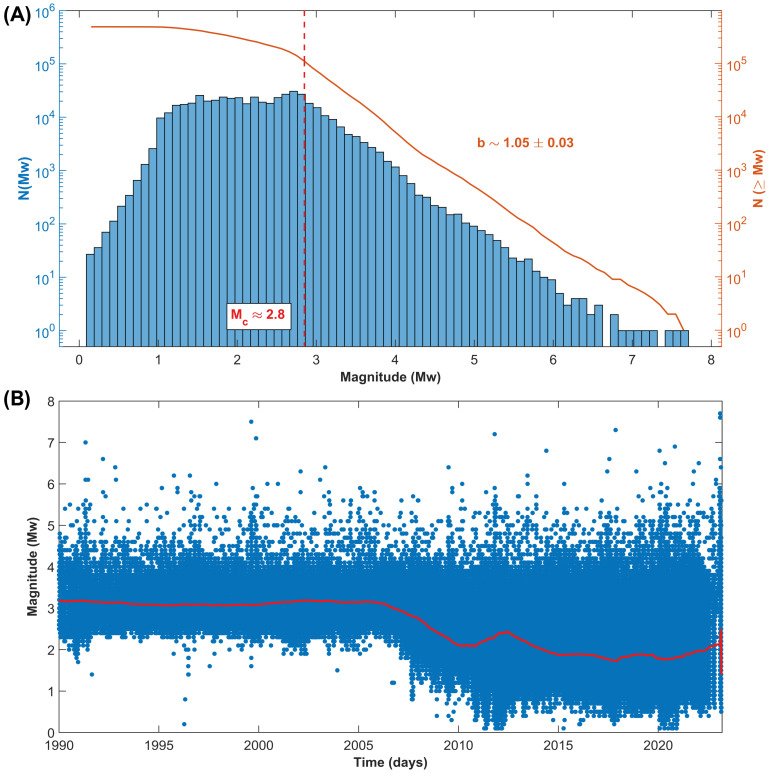
(**A**) Frequency-size distribution of the shallow Turkish seismicity (1990–2023, longitude 25–46${}^{\circ }$ E and latitude 34–44${}^{\circ }$ N, and hypocenters shallower than 30 km). (**B**) Catalogue completeness from 1990 to 2023. The red line represents the smoothed completeness magnitude calculated using samples of one thousand earthquakes each.

More than one hundred thousand earthquakes have been recorded above the completeness magnitude, whose average value has been estimated to be about M${}_{c}∼$ 2.8, which decreased from about ∼3.3 in 1990 to less than 2.0 currently (compare with Figure 2) because of the increase in the number of AFAD and KOERI stations after the 1999 Izmit and Düzce earthquakes.

**Figure 3:**
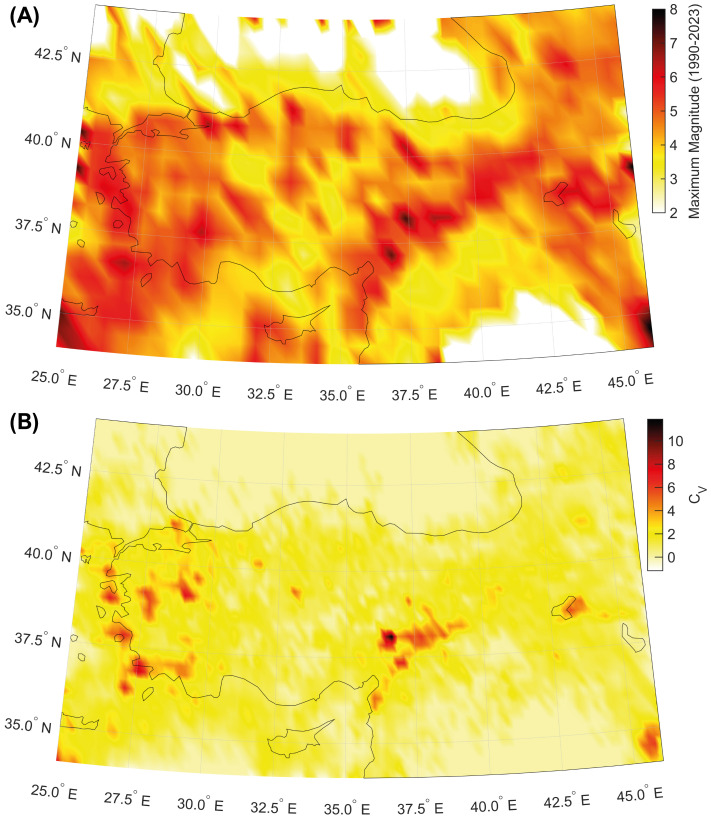
(**A**) Map of the maximum magnitude in the catalogue. (**B**) Map of the global coefficient of variation C${}_{V}$ of inter-event times (seismic events occurring in the period 1 January 1990–27 February 2023, longitude 25–46${}^{\circ }$ E, latitude 34–44${}^{\circ }$ N, and hypocenters shallower than 30 km, are considered).

The largest seismic events of the last three decades occurred in the Kahramanmarş region, being the 6th February 2023 M${}_{w}$ 7.8 and 7.6 seismic doublet [33] and the M${}_{w}$ 7.4 17 August 1999 Izmit earthquake [34]. Both areas are characterized by high values of the global coefficient of variation. This peculiarity is also shared with other zones along the western Aegean coast of Turkey, which is also prone to large seismic events (Figure 3).

**Figure 4:**
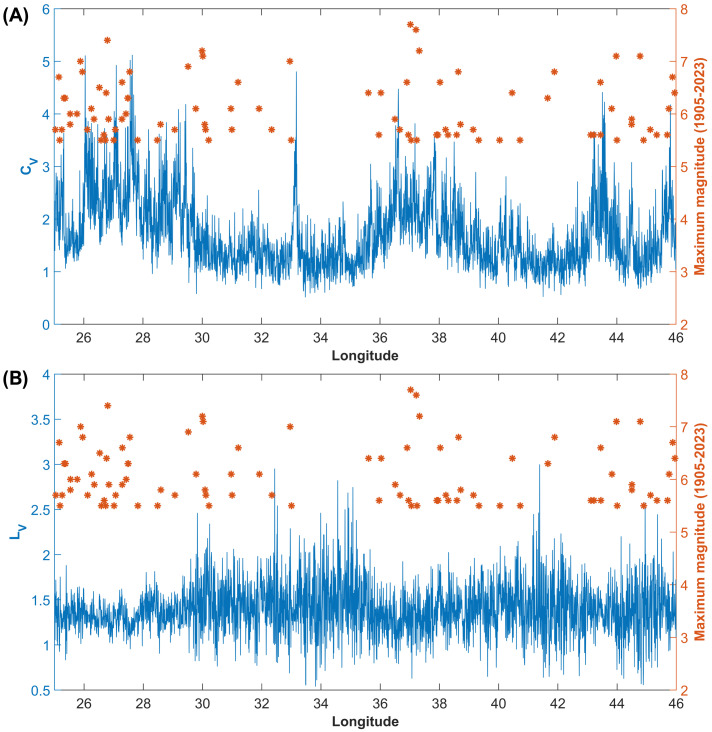
Spatial distribution of the global C${}_{V}$ (**A**) and local L${}_{V}$ (**B**) coefficient of variation of inter-event times. The blue line represents the C${}_{V}$ for seismicity reported in the TURHEC catalogue from 1990 to 2023, while the orange asterisks stand for the large (M${}_{w}$ 5.5+) recorded in Turkey since 1905.

Moreover, a comparative analysis of the spatial distribution of $C_{V}$ and $L_{V}$ estimated using data from the TURHEC (1990–2023) shows that the largest seismic events from 1905 to 2023 were nucleated in regions hosting globally clustered and locally Poissonian seismicity (Figure 4).

**Figure 5:**
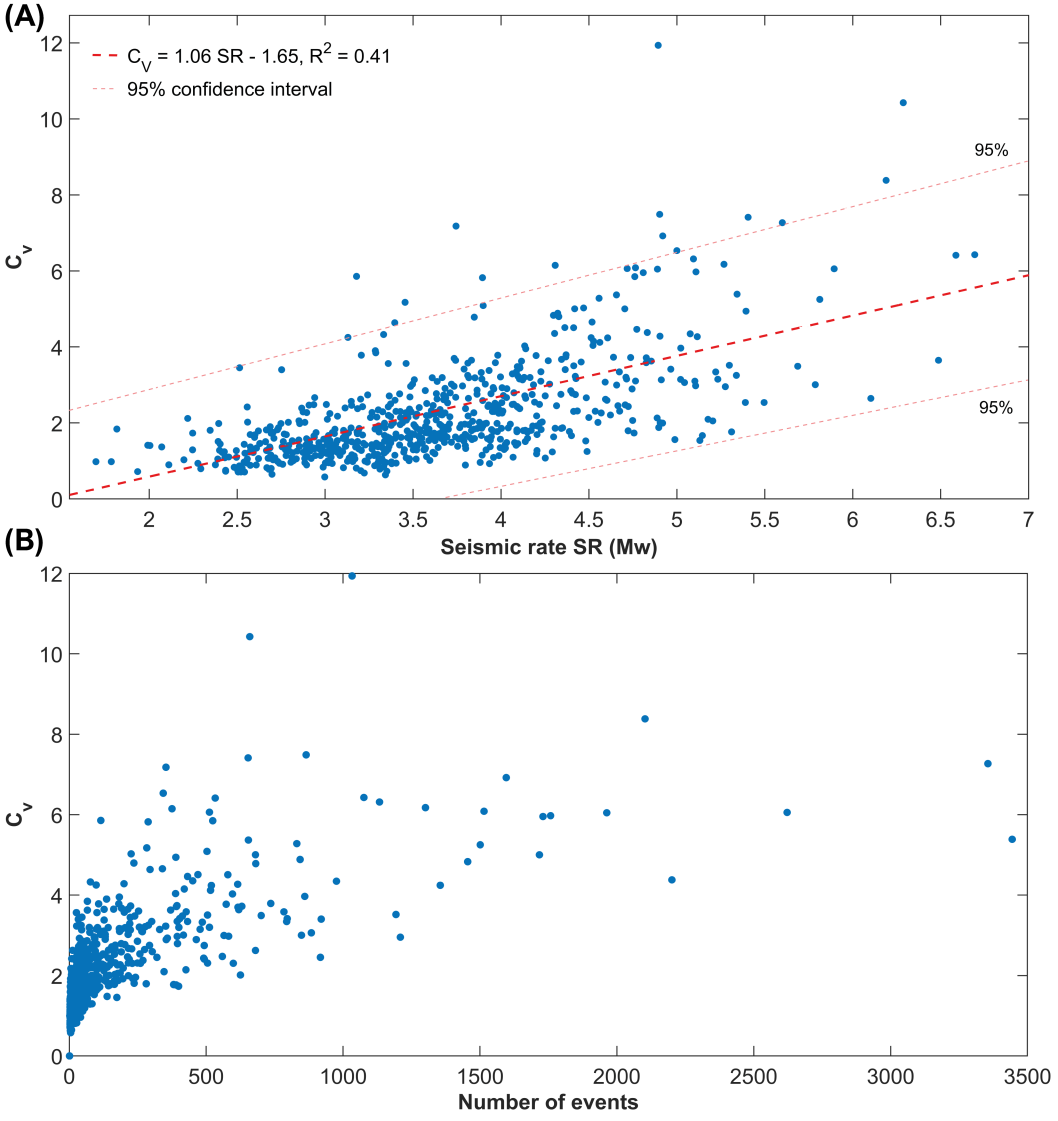
(**A**) Correlation between the global coefficient of variation C${}_{V}$ and the annual seismic rate inferred using the regional seismic activity above the completeness magnitude from 1990 to 2023. The linear fit is represented by the dashed thick red line, while the 0.95 prediction intervals are marked by the dashed pink thin ones. (**B**) The global coefficient of variation is weakly positively related to the length of the seismic catalogue. For large seismic catalogues (≥1000 events), C${}_{V}$ appears to be almost independent of the number of recordings.

In addition, a positive correlation has been observed between the global clustering coefficient of the inter-event times and the local seismic rate, defined as the annual amount of energy nucleated by seismicity in the selected area, expressed as a moment magnitude equivalent, and the number of events. For the sake of simplicity, we use a linear fit, as the data is too scattered to apply more complex functions; however, the coefficient of variation is a positive number and the linear relationship is to be considered within the range of magnitudes constrained by the observations. The second trend shows a roughly logarithmic dependence of C${}_{V}$ on the number of earthquakes *N*, so that, while for small subsets (N ≤ 500) an almost linear relationship exists between the two parameters, for large datasets (N ≥ 1000), the size effect is almost negligible (Figure 5).

**Figure 6:**
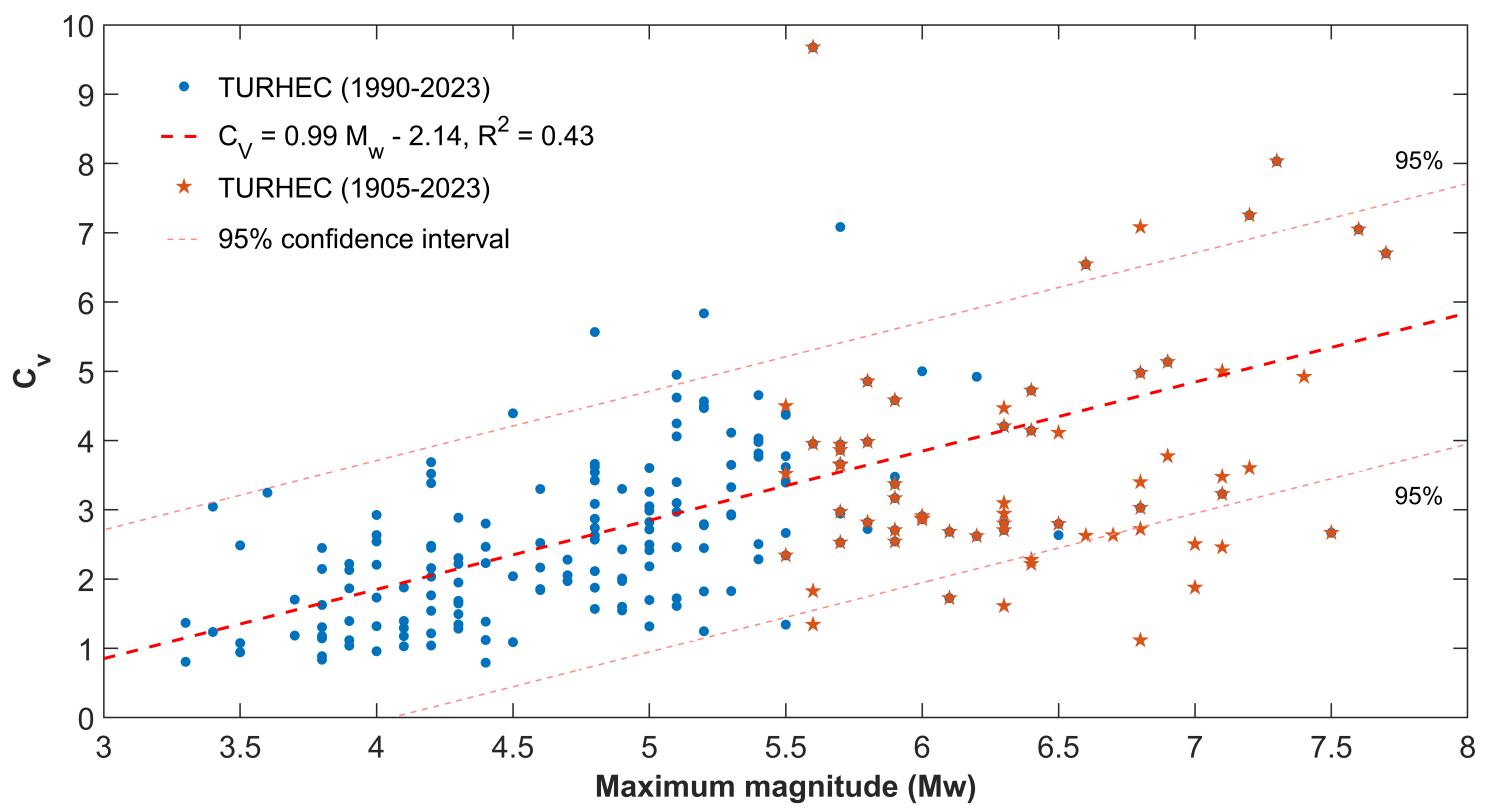
The global coefficient of variation is positively correlated with the maximum magnitude in catalogue. The blue circles represent the C${}_{V}$ for each spatial grid element; we segmented the whole region (30 × 15) with at least one hundred seismic events in the catalogue (occurring in the period 1 January 1990–27 February 2023, longitude 25–46${}^{\circ }$ E, latitude 34–44${}^{\circ }$ N, and hypocenter shallower than 30 km). Orange stars stand for the largest earthquakes (≥M${}_{w}$ 5.5) occurring since 1905 in each segment. The linear fit is represented by the dashed thick red line, while the pink thin ones mark the 0.95 prediction intervals.

Even clearer is the correlation between the global coefficient of variation of the inter-event times and the maximum magnitude observed in the catalogue (1990–2023). Surprisingly, the statistical trend is still observed considering the maximum magnitude listed in the whole TURHEC, which supports the output already reported in Figure 4. Compare with Figure 6: the blue dots represent the global coefficient of variation of seismicity from 1990 to 2023 in each investigated region as a function of the maximum observed magnitude, while the orange stars mark the same in the case of the TURHEC catalogue since 1905 (M${}_{w}$≥ 5.5). It is worth noting that the blue points tend to be located above the dashed red fit line; in contrast, the stars (except for an outlier) are mainly below the dashed line. A possible interpretation is that regions where higher values of the coefficient of variation of the inter-event times are observed, determined by the size of the largest seismic event in catalogue, are likely to be more prone to hosting major earthquakes in the future with respect to regions characterized by lower values. So, it might just be a matter of time before the next large event. This hypothesis is consistent with what is shown in Figure 4 and Figure 5.

**Figure 7:**
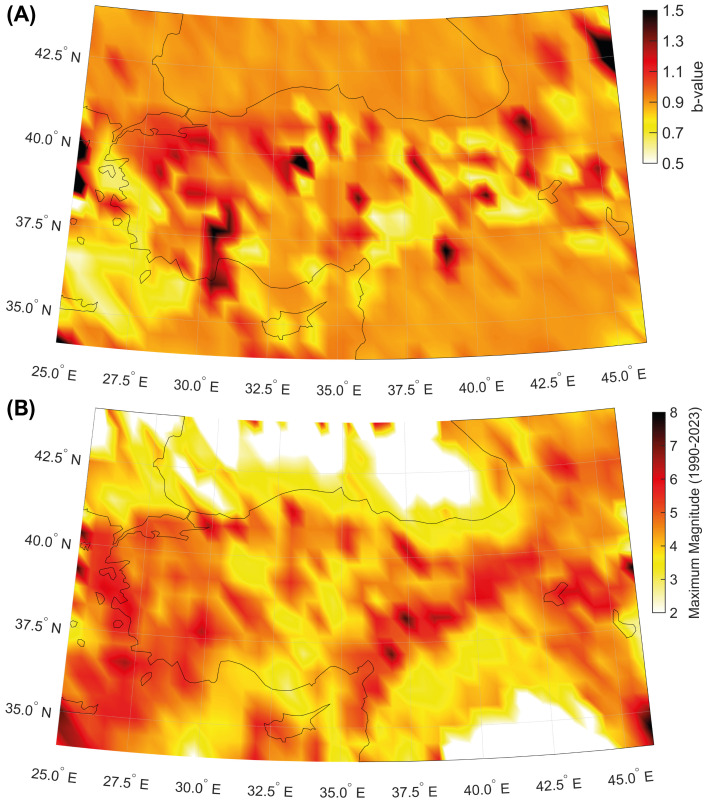
(**A**) Map of the b-values of the Gutenberg–Richter scaling law (seismic events occurring in the period 1 January 1990–27 February 2023, longitude 25–46${}^{\circ }$ E, latitude 34–44${}^{\circ }$ N, and hypocenter shallower than 30 km, are considered). (**B**) Map of the maximum magnitude in the catalogue.

In our study, we also investigate the spatial and temporal distribution of the b-values of the Gutenberg–Richter law. We find some regions with lower values of the scaling exponents located along the Northern Anatolian and, above all, along the Eastern Anatolian fault system and offshore of the western coasts of Turkey. A zone with an apparently low b-value is also observed close to the Karliova Triple Junction. Higher values are located in the central and western part of the country between longitude 28 and 31${}^{\circ }$ E. We identify a negative correlation with the maximum magnitude in the TURHEC catalogue (shallow crustal events from 1990 to 2023). Compare with Figure 7.

**Figure 8:**
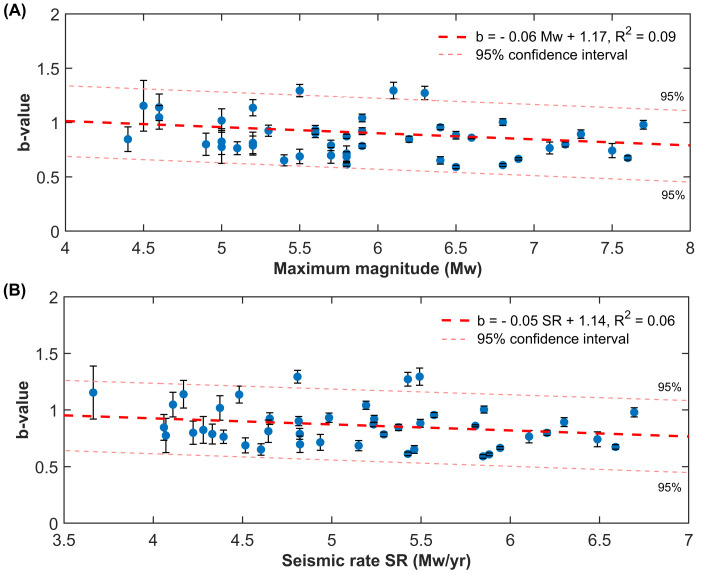
A negative trend is observed between the b-value and the annual seismic rate (**A**) and the maximum magnitude in the catalogue (**B**) in Turkey (seismic events occurring in the period 1January 1990–28 February 2023, longitude 25–46${}^{\circ }$ E, latitude 34–44${}^{\circ }$ N, and hypocenter shallower than 30 km, are considered). The Anatolian region is segmented using a rectangular grid (6 × 15 elements); the b-value is included in the plots above provided that at least 300 earthquakes are reported within each region. The error bars stand for the 2σ uncertainty confidence intervals and the dashed pink thin lines represent the 95% confidence intervals for the linear fit (in dashed thick red).

A more quantitative analysis shows a negative relationship between the b-values of the Gutenberg–Richter law and the seismic rate and the maximum magnitude. However, the negative trend between the scaling exponent of the frequency-size distribution and the amount of annual nucleated energy within sub-regions of a looser grid (see the Section 2), although statistically significant, shows large residuals with respect to the linear trend. The reason is that the uncertainties of the b-values are quite small; so, the R${}^{2}$ is extremely low, which means that the data variability cannot be explained just by the linear relationship used for fitting our data. Compare with Figure 8. The same result is found in the case of the maximum magnitudes. A possible explanation is that the b-value is investigated across the entire Anatolian region where a mixture of different tectonic settings exists and a large variation in crustal states of stress takes place and a simple linear fit is not able to take into account such local effects.

**Figure 9:**
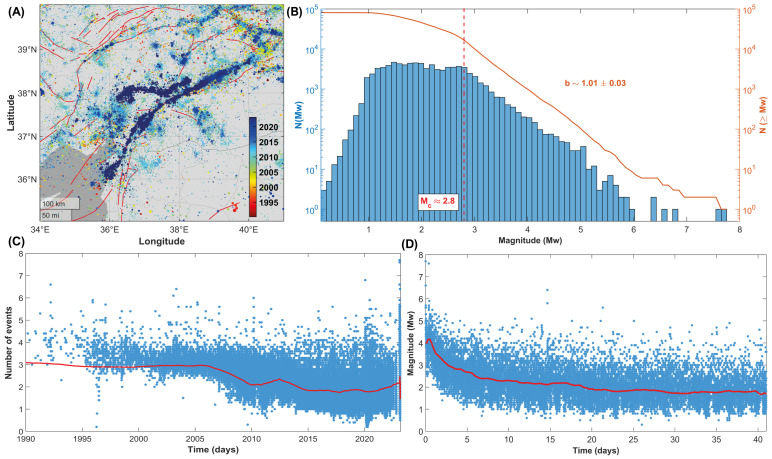
(**A**) Map of seismicity in the Kahramanmaraş region from 1990 to 2023 (events with longitude 34–41${}^{\circ }$ E, latitude 35–40${}^{\circ }$ N, and hypocenter shallower than 30 km, are considered). (**B**) Frequency-size distribution of seismicity. Blue bars represent the probability density function, while the orange line stands for the cumulative magnitude distribution. (**C**) Catalogue completeness from 1990 to 2023 and from 6 February 2023 to 19 March 2023 (**D**). The red line is the smoothed completeness magnitude calculated using samples of one thousand seismic events each.

In the second part of our investigation, we focus on the Kahramanmaraş region and the seismic sequence still ongoing there. Figure 9 represents seismicity from 1990 to 2023 (events with longitude 34–41${}^{\circ }$ E, latitude 35–40${}^{\circ }$ N, and hypocenter shallower than 30 km are plotted), considering the Gutenberg–Richter law (orange line, Figure 9B) and the density distribution of magnitudes (blue bars). In the lower plots (Figure 9C,D), the temporal evolution of the completeness magnitude is shown.

**Figure 10:**
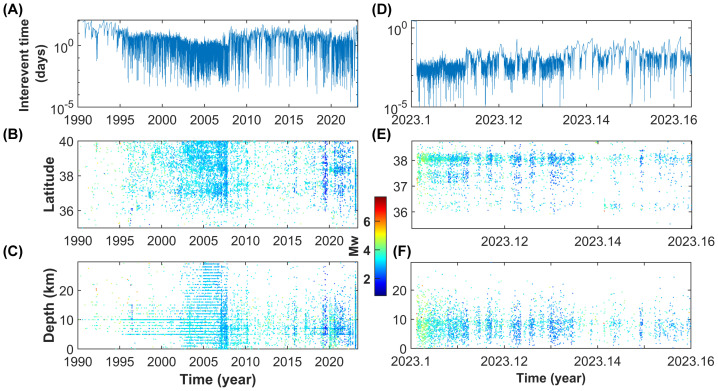
Spatial and temporal distribution of seismicity in the Kahramanmaraş region. The plots (**A**–**C**) show the outputs relative to the whole period of investigation (1990–2023); (**D**–**F**) present the results for the Kahramanmaraş seismic sequence. The upper panels represent the inter-event time, while the mid and lower plots show how the seismicity above the completeness magnitude is distributed in latitude and depth.

We analyze the spatial distribution of seismicity above the completeness magnitude since 1990 and the inter-event times (Figure 10). Although from 1990 to 2010 a decreasing trend in the duration of the inter-event intervals is observed because of the progressive lowering of the completeness magnitude due to improvements in the seismic network, a slow, but significant, acceleration in seismic activity is detected since 2014. This evolution led to the M${}_{w}$ 6.7 Do$\overset{˘}{g}$anyol which occurred on 24 January 2020 and culminated just after the Kahramanmaraş seismic doublet on 4 February 2023. The decrease in the inter-event times from 2014 to 2020 is mainly due to seismic events located at a depth of 10–25 km distributed along almost all the considered faulting region, without the occurrence of any sizeable spatial cluster.

**Figure 11:**
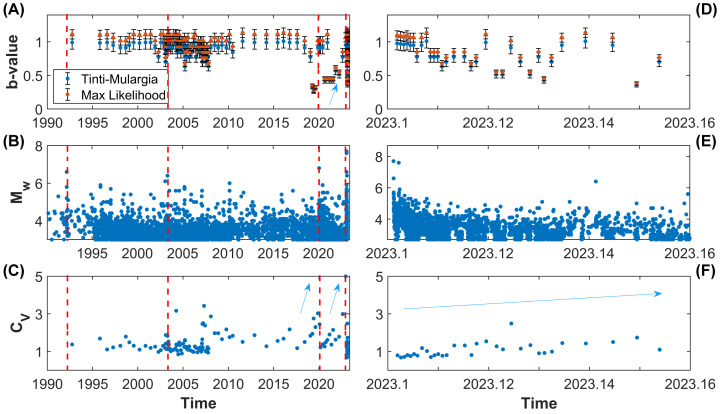
A comparison of the statistical and clustering properties of seismicity in the Kahramanmaraş region from 1990 to 2023 (**A**–**C**) and since 6 February 2023 (**D**–**F**). In the upper plots, the variations in the b-values of the Gutenberg–Richter law are reported. The b-value is estimated using two different techniques (the Tinti–Mulargia method—blue points—and Aki’s maximum likelihood method—orange circles). Seismicity above M${}_{w}$ 3.0 is shown in the central plots, while the global clustering coefficient is plotted below.

Jointly with the inter-event time and the global coefficient of variation, the temporal changes in the b-value of the Gutenberg–Richter law are usually estimated, providing insightful information about the dynamics preceding large seismic events. Therefore, even in this work, we report the temporal variation in the scaling exponent of the frequency-size law of earthquakes above the completeness magnitude in the investigated area. Figure 11 shows that the large 2020 and 2023 earthquakes are forewarned by a several-months-long drop in the b-value, as well as by an increase in the global coefficient of variation of the inter-event times, C${}_{V}$ (see Figure 11A–C). The decrease in the b-value from about 1.0 to 0.4 started during the second half of 2018. A progressive increase is observed after the M${}_{w}$ 6.7 earthquake, but its values have never returned to their previous level, with further fluctuations occurring during the Kahramanmaraş seismic sequence. The variations in the b-value are also accompanied by changes in C${}_{V}$; in our case, an accelerated increase is observed both before the 2020 and the 2023 seismic sequences. It may suggest that seismicity tends to cluster before major events in this region. A real physical effect in the change in the b-value is likely, even more so in light of the concomitant variations in the clustering properties. Nevertheless, non-physical contributions might play a role in reducing its value, for instance, because of rapid changes in the magnitude completeness that our analysis (based on groups of several hundred events each to provide better estimates of the scaling exponent of the Gutenberg–Richter law) does not have the resolution to highlight.

## 4. Discussion

### 4.1. Seismotectonic Context and Historical Seismicity

The Anatolian Plate is located in a quite complex geodynamic setting, at the boundary between the African, Arabian and Eurasian Plates. Extended GNSS time surveys show that the Anatolian Plate undergoes a counter-clockwise rotation [35]. Moreover, a motion ranging between 18 and 28 mm/year is recorded by geodetic stations across the North Anatolian Fault and the Marmara Sea, e.g., [36]. Along the northern transform boundary near the Black Sea coast, as well as along the East Anatolian Fault, frequent seismic activity is recorded. Being prone to major seismic events and densely populated in some areas, great attention has been paid by the scientific community to improve the hazard assessment of this region (e.g., [37,38,39,40]). In addition, the Aegean area hosts large earthquakes. Therefore, except for a small portion of its territory, mainly located in the inner regions, Turkey is an extremely active earthquake and volcanic region [41] (Figure 1). Turkey has been hit by several large events. The 17 August 1668 North Anatolia earthquake (M${}_{w}$ 7.8–8.0) [42] was likely the largest known. More recent sequences followed the devastating 26 December 1939 Erzincan M${}_{w}$ 7.9 quake [43], which likely produced the further destabilization which was the reason for the 1942–1944 seismic activity, continuing with major quakes in 1949, 1951, 1957, 1966–1967, 1992 and two in 1999. The last one culminated with the 17 August 1999 M${}_{w}$ 7.4 Izmit earthquake [44] and the following 12 November 1999 M${}_{w}$ 7.2 Düzce event [45]. The Aegean region also nucleated large seismic events, such as the 23 July 1949 Chios [46], the 24 April 1957 M${}_{w}$ 7.1 Ortaca, and the 30 October 2020 M${}_{w}$ 7.0 Izmir Bay earthquake [47]. Large seismic events also occur in intraplate Turkish territories, such as in the case of the 28 March 1970 Gediz M${}_{w}$ 7.0 earthquake [48]. More recently, south-eastern Turkey and its neighboring areas of Syria have been hit by the largest seismic events ever reported in regional instrumental catalogues. On 6 February 2023 at 01:17:35 UTC, a M${}_{w}$ 7.8 strike-slip faulting earthquake involved the East Anatolian Fault. After nine hours, the main shock was followed by a M${}_{w}$ 7.6 twin earthquake nucleated by the Sürgü fault at 10:24:49 UTC about 150 km to the north-west [33]. Thanks to the recent enhancement of the regional KOERI and AFAD seismic networks [49] (compare with the progressive lowering of the completeness magnitude in Figure 2) and the publication of homogeneous catalogues [25] since the occurrence of the 1999 Izmit event, it is now possible to perform an advanced statistical and clustering analysis covering more than thirty years of recordings.

### 4.2. Clustering and Scaling Properties of Turkish Seismicity and Its Regional Variability

Our analysis shows that the largest seismic events in Turkey occur in regions where seismicity is featured by locally Poissonian and globally clustered behavior (Figure 3B and Figure 4). The maximum magnitudes in the catalogue since 1905 are also positively correlated with the global coefficient of variation, calculated using available seismicity data from events occurring during the last three decades (Figure 5 and Figure 6). Our results are in agreement with preceding recently published research [23,50]. Moreover, the spatial mapping of the scaling exponent of the Gutenberg–Richter law also provides interesting information. This analysis is performed by taking advantage of the events above the completeness magnitude listed in the TURHEC and which occurred from 1 January 1990 to 27 February 2023 from latitude 34${}^{\circ }$ to 44${}^{\circ }$ N and between longitude 25${}^{\circ }$ and 46${}^{\circ }$ E, with hypocentral depth shallower than 30 km. We divide the Anatolian region into rectangular contiguous areas. For the assessment of the b-value, a 15 × 6 grid, along longitude and latitude, respectively, is utilized in order to guarantee reliable statistical results. See Figure 7 and Figure 8. Regions where seismicity is characterized by lower b-values than the surrounding areas are identified along a large part of the East Anatolian fault system, while isolated spots are observed along the North Anatolian transcurrent boundary. The Aegean coast and sea is also characterized by low b-values. Conversely, the northwestern part of Turkey, as well as the Antalya area, host seismic activity with rather high b-values. A negative correlation between the local b-value, the seismic rate, and the maximum magnitude is observed (Figure 8).

### 4.3. Seismic Activity in the Kahramanmaraş Region

Our analysis focussing on the Kahramanmaraş region confirms the results discussed above and also highlights an anomalous drop in the b-value since 2018 (from 1.0 to b ∼ 0.4) in the region shaken by the 2023 seismic sequence, accompanied by significant changes in the global coefficient of variation (Figure 11). The value of the scaling exponent of the Gutenberg–Richter law appears to recover its equilibrium condition (b ≈ 0.9–1.0) after the occurrence of the earthquake doublet on 6 February 2023, even though fluctuations are still observed with an apparent long-term decrease. This evidence is consistent with other peculiar seismological patterns recently reported in scientific publications, e.g., [51]. Our results suggest a progressive acceleration of seismic activity in the region since 2018 with a first peak reaching to the north in January 2020, corresponding to the Do$\overset{˘}{g}$anyol 2020 seismic sequence, which, probably, produced a further destabilization in the southern area, subsequently hit by the Kahramanmaraş events.

### 4.4. Implications and Physical Interpretation

Our research clearly shows that a relationship exists between the clustering and statistical properties of seismicity in Turkey. Large seismic events tend to occur where small to moderate activity is featured by locally Poissonian and globally clustered behavior, low b-values, and an elevated seismic rate. As suggested in [23], such a connection may arise from the mechanism of stress accumulation and release as a function of the structural complexity, fault roughness and rheological heterogeneity of fault systems, e.g., [52,53,54,55]. A mechanically weak interface is characterized by low internal friction, so it cannot hold high spatial stress concentration, producing diffuse small magnitude seismicity along the interface; conversely, strong faults enhance stress accumulation and, therefore, the probability of large seismic events increases. Frequent strain release seems to be associated with diffuse, globally Poissonian seismicity with mid-to-high b-values and a relatively low maximum magnitude; in contrast, where fault systems are completely locked, small events occur clustered in time and space, usually organized in swarms or short seismic sequences. Cascade triggering processes are ultimately responsible for larger seismic events, which play a crucial role in re-establishing the mechanical stability of the whole fault system, and also producing significant stress drop. In summary, the geophysical properties of the crustal volumes and major faults, fracturing, statistical features, and clustering of seismicity, are closely connected to each other and to the regional seismogenic potential.

## 5. Conclusions

In this study we perform a clustering analysis of seismicity in Turkey, paying special attention to the Eastern Anatolian region recently hit by the M${}_{w}$ 7.8 and 7.6 seismic doublet, followed by widespread aftershocks. Our results suggest that large earthquakes are more likely to occur in zones characterized by globally clustered, locally Poissonian seismicity, and low b-values. A clear positive correlation is observed between $C_{V}$ and the annual seismic rate (Figure 5) and the maximum magnitude in catalogue (1990–2023). The effect is still observed when comparing the clustering properties with large seismic events recorded over longer time periods (1905–2023) (see Figure 4 and Figure 6). The prediction intervals and the goodness-of-fit confirm that our conclusions are supported by statistical analysis. Regions with higher values of the global coefficient of variation of inter-event times, C${}_{V}$, are likely to be more prone to nucleating large earthquakes in the near future than regions characterized by lower values, if their largest seismic events have the same magnitude. We think that new studies are required in order to understand to what extent such effects are common to, and statistically significant in, different tectonic regions, having already been observed in New Zealand [23]. If our hypothesis is confirmed, the clustering properties should be considered as a possible additional information source for the assessment of seismic hazard. We also highlight significant variations in both the b-values and the global coefficient of variation of inter-event time series before the largest seismic events in the Kahramanmaraş region, suggesting accelerated energy release and foreshock activity. The result is verified using two different methods for the estimation of the frequency-size scaling exponent, as shown in Figure 11.

## Data Availability

The updated version of the Turkish Homogenized Earthquake Catalogue (TURHEC), first version available at https://doi.org/10.5281/zenodo.5056801, can be obtained by reasonable request from Onur Tan (onur.tan@iuc.edu.tr), last accessed on 27 February 2023 for the present work; while the catalogue of aftershocks is available on the AFAD portal (https://deprem.afad.gov.tr/event-catalog, accessed on 27 February 2023). The maps in the article were realized using the Matlab mapping toolbox and the Global Active Faults database available at https://github.com/GEMScienceTools/gem-global-active-faults (last access on 20 March 2023).
